# Systematic map of recent evidence on reproductive performance of cattle in Africa

**DOI:** 10.1007/s11250-024-04074-z

**Published:** 2024-07-22

**Authors:** Fiona K. Allan, Isla S. MacVicar, Andrew R. Peters, Christian Schnier

**Affiliations:** Centre for Supporting Evidence Based Interventions-Livestock (SEBI-Livestock), Royal (Dick) School of Veterinary Studies, University of Edinburgh, Easter Bush Campus, Midlothian, EH25 9RG UK

**Keywords:** Africa, Cattle, Fertility, Reproduction, Systematic mapping

## Abstract

Good cattle reproductive performance is essential for livestock productivity. Farmers are highly dependent on the success of productive outputs to support their livelihoods. In low- and middle-income countries (LMICs), however, optimal reproduction of cows and bulls is comparatively less well defined, with information on key reproductive parameters and reproductive management often not available. The aim of this study was to collate and synthesise the recent published evidence on cattle reproductive performance in selected sub-Saharan countries.

Systematic mapping methodology was used, with searches conducted for both cow and bull reproductive performance in Eritrea, Ethiopia, Kenya, Nigeria, Somalia, South Sudan, Sudan, Tanzania and Uganda, for the period 2012–2022, in English language. Search returns were screened for relevance at title and abstract, and full-text levels, based on the research question criteria.

A substantial number of studies were identified for cows (*n* = 133), but only very few for bulls (*n* = 11). A large proportion of reported studies have been conducted in Ethiopia, with relatively few from the other countries, and most studies published between 2014 and 2016, and in 2021. Certain reproductive parameters received more attention than others; calving interval was reported in 86 studies, while culling due to infertility was reported in eight studies.

The study highlights where research is being conducted in this area, and importantly where there is a gap, in particular on bull reproductive performance. While there were a range of values reported for cow reproductive parameters, the values were reasonable, indicating that it is possible to have good reproductive performance in LMICs. The synthesis of studies in the map should help to inform farmers and their advisors, at farm and national levels.

**Supplementary Information:**

The online version contains supplementary material available at 10.1007/s11250-024-04074-z.

## Introduction

A high level of fertility in cattle, generally referred to as reproductive performance, is important for efficient and successful livestock productivity and economic impact (Diskin 2011). Cattle reproductive performance is intrinsically linked to overall productivity through milk yield, meat and calf production (Ball and Peters 2004b). Thus, these productive outputs impact all livestock farmers but in particular the overall food security, nutrition and income of pastoral and small-holder farmers in low-and middle-income countries (LMICs) (Smith et al. 2013; Tona 2021). Moreover, without the continual successful production of offspring, there is reduced capacity for the replacement of livestock and for the genetic improvement of a herd (Ball and Peters 2004b). Farmers in LMICs are highly dependent on the success of these productive outputs to support their livelihoods, and to supply increasing demand (Moran 2005). In general, reproductive performance of cattle in Africa is thought to be low (Ouma et al. 2007), however there have been no recent reviews of cattle fertility in the region.

In order to optimize cattle reproductive performance, farmers and their advisers need information about key reproductive parameters. In modern production systems, a dairy cow optimally calves for the first time at approximately 24 months of age, and thereafter produces one healthy calf every 12 months i.e. a calving interval of 12 months. Ideally this is achieved with few inseminations per conception and with a low number of cows being culled for infertility (Ball and Peters 2004b; Webster 2020). A dairy bull optimally produces a consistently high amount of healthy sperm, and has the physical fitness and libido to achieve intromission and deposit semen in the vagina (Penny 2009). Bull fertility is currently phenotypically evaluated with a breeding soundness examination (BSE) which includes an examination of sperm for numbers, motility and abnormalities (Butler et al. 2020). Under more traditional production systems in LMICs, optimal reproductive performance of cows and bulls is comparatively less well defined, with information on key reproductive parameters and reproductive management often unavailable (Abin et al. 2018). In LMICs, resources, funding, or insufficient training can all contribute to limitations in research.

Cattle reproductive performance is influenced by various factors; internal (animal factors), external, and management factors. Animal factors, including egg and sperm health, hormonal patterns and stress, have an effect on reproductive performance (Evenson 1999). Age and breed traits, such as the ability to withstand certain stressors, can impact the reproductive success of a herd (Cielava et al. 2017). Indigenous breeds are often selected for their ability to thrive in their local environment, whereas exotic breeds tend to be utilised to improve overall production (Pryce et al. 2004). Nutritional status and disease burden can affect the reproductive performance of cattle during pregnancy as well as post-partum, which can impact the success of future conception (Ciccioli et al. 2003; Sulieman et al. 2017). External factors such as climate, season and agro-ecological zones influence reproductive performance and their impact can be challenging to navigate (De Kruif 1978).

Farmers and the livestock sector in general need to understand reproductive performance at both farm level and at national level. Improving the reproductive performance of livestock in LMICs is often cited as an objective in national Livestock Master Plans (for example, Shapiro et al. 2015; Michael et al. 2018), yet LMICs often lack the evidence base required for such planning (Schelling et al. 2007). For government decision-makers to be able to successfully implement interventions for improvement, an evidence base of reproductive performance is needed, including performance measurements. Systematic evidence mapping methodology extracts meta-data that describes the type and quantity of research in a specific field, as opposed to the research findings themselves (James et al. 2016). As systematic maps can address broad, multi-faceted questions, they can cover the breadth of scientific information needed for decision-making and policy-based questions (Dicks et al. 2014). While mapping of literature is not intended to replace a systematic analysis of factors associated with reproductive performance, such as association of breed or a region with reproductive performance, systematic mapping is very useful to map out the differences in reproductive performance that have been studied in different regions. The objective of the current work was to systematically identify and map the evidence of cattle reproductive performance in selected sub-Saharan African countries, to highlight both the gluts and gaps of information to support and improve evidence-based decision-making for farmers and their advisers.

## Materials and methods

### Research question

The research question that formed the basis for the systematic map was:

- What is the recent available evidence on cattle reproductive performance in Eritrea, Ethiopia, Kenya, Nigeria, Somalia, South Sudan, Sudan, Tanzania and Uganda?

(See Study screening and selection, below, for justification of these inclusion criteria).

The key elements of the research question were formulated following the Population and Outcome (PO) scheme (Liberati et al. 2009):

- Population: Cattle.
- Outcome: Distribution of evidence, cattle reproductive performance.

### Protocol

Planned methodology was documented a priori in a protocol detailing the proposed approaches for the systematic map. In the absence of a standard protocol format for the registration of animal study systematic reviews (de Vries et al. 2015), a protocol format developed by de Vries et al. (de Vries et al. 2015), based on the Cochrane review protocol (Green and Higgins 2011) and the PRISMA (preferred reporting items for systematic reviews and meta-analyses) checklist (Page et al. 2021), was used for this review. This standardised protocol format is supported by CAMARADES (Collaborative Approach to Meta Analysis and Review of Animal Data from Experimental Studies) and SYRCLE (SYstematic Review Center for Laboratory animal Experimentation), the largest supporting groups involved in systematic review of data from animal studies. The protocols were completed early in the review process and were approved by all authors before prospectively registering the protocols online (Female cattle: 10.7488/era/2910; Bulls: https://www.research.ed.ac.uk/en/publications/protocol-for-systematic-map-of-reproductive-performance-of-male-c). The PRISMA checklist is also included, in Supplementary File S1. This systematic map did not require ethical approval.

### Information sources

A variety of electronic databases, search engines and websites were used to identify published and grey literature. Bibliographic databases used were Web of Science, Scopus, PubMed (Medline) and CAB Direct. These databases are considered to cover the majority of veterinary literature (Grindlay et al. 2012). Search engines and websites included Google Scholar, Gates Open Research, Research4life, CGIAR and Global ETD (Networked Digital Library of Theses and Dissertations archive). The potential for non-reporting bias was minimised by consulting multiple databases.

### Scoping review

A preliminary search was carried out in June-July 2022 to test search terms and strings, with commonly used terminology, and to gauge the volume of available evidence. Search terms were reformatted and reassessed until a consensus was achieved.

### Search strategy

Literature searches were carried out for all included years (2012–2022 inclusive), in the selected databases, in July 2022 for cows and in May 2023 for bulls.

Boolean search strings in the form of (Population) AND (Outcome) were developed for each database, including AND (countries), AND (publication years) where appropriate. Details of the terms used and the search strings in each of the searches for cows and bulls are provided in Tables 1 and 2, respectively. The respective search strings were tested in selected databases initially and were adapted by the authors until finalised. Literature searches for both cows and bulls were restricted on publication dates from 2012 to 2022 and the number of studies retrieved by each search string were recorded. Due to the project’s restricted timeline, snowballing (using reference lists to identify additional papers) was not performed.

**Table 1:** – Results for each database and search string for female cattle fertility. For each search string, the first number of results is the number of returns identified; the second number is results after de-duplication

Database source	Search string	Number of results
Web of Science	(TS = (Reproduction) OR TS = (Fertility) OR TS = (reproductive) OR TS = ("days open") OR TS = ("calving age") OR TS = ("calving interval") OR TS = ("days open") OR TS = ("calving age") OR TS = ("breeding age") OR TS = ("non-return") OR TS = ("conception rate") OR TS = ("age at first calving") OR TS = ("calving interval")]) AND ( TS = (Ethiopia) OR TS = (Tanzania) OR TS = (Kenya) OR TS = (Nigeria) OR TS = (Sudan) OR TS = (Somalia) OR TS = (Eritrea) OR TS = (Uganda)) AND (TS = (Cow) OR TS = (cattle) Or TS = (bos)) AND PY = (2011–2022) NOT (TS = (soil) OR TS = (trial))	210 (209)
Scopus	(TITLE-ABS-KEY(Reproduction) OR TITLE-ABS-KEY(Fertility) OR TITLE-ABS-KEY(reproductive) OR TITLE-ABS-KEY("days open") OR TITLE-ABS-KEY("calving age")OR TITLE-ABS-KEY("calving interval") OR TITLE-ABS-KEY("days open") OR TITLE-ABS-KEY("calving age") OR TITLE-ABS-KEY("breeding age") OR TITLE-ABS-KEY("non-return") OR TITLE-ABS-KEY("conception rate") OR TITLE-ABS-KEY("age at first calving") OR TITLE-ABS-KEY("calving interval")]) AND ( TITLE-ABS-KEY(Ethiopia) OR TITLE-ABS-KEY(Tanzania) OR TITLE-ABS-KEY(Kenya) OR TITLE-ABS-KEY(Nigeria) OR TITLE-ABS-KEY(Sudan) OR TITLE-ABS-KEY(Somalia) OR TITLE-ABS-KEY(Eritrea) OR TITLE-ABS-KEY(Uganda)) AND (TITLE-ABS-KEY(Cow) OR TITLE-ABS-KEY(cattle) Or TITLE-ABS-KEY(bos)) AND PUBYEAR > 2011 AND NOT (TITLE-ABS-KEY(soil) OR TITLE-ABS-KEY(trial))	232 (78)
Google Scholar	(Cow OR cattle OR bos) AND (Reproduction OR reproductive OR fertility) [title words] ((Ethiopia OR Tanzania OR Kenya OR Nigeria OR Sudan OR Somalia OR Uganda OR Eritrea)) [key words]	405 (346)
CGIAR	https://cgspace.cgiar.org/discover?filtertype_1=title&filter_relational_operator_1=contains&filter_1=Cow&filtertype_2=title&filter_relational_operator_2=contains&filter_2=reproduction&query=(Reproduction+OR+Fertility)+AND+(Ethiopia+OR+Tanzania+OR+Kenya+OR+Nigeria+OR+Sudan+OR+Somalia+OR+Eritrea+OR+Uganda)+AND+(Cow+OR+cattle)&scope = / https://cgspace.cgiar.org/discover?filtertype_1=title&filter_relational_operator_1=contains&filter_1=Cow&filtertype_2=title&filter_relational_operator_2=contains&filter_2=reproductive&query=(Reproduction+OR+Fertility)+AND+(Ethiopia+OR+Tanzania+OR+Kenya+OR+Nigeria+OR+Sudan+OR+Somalia+OR+Eritrea+OR+Uganda))+AND+(Cow+OR+cattle)&scope = / https://cgspace.cgiar.org/discover?filtertype_1=title&filter_relational_operator_1=contains&filter_1=Cow&filtertype_2=title&filter_relational_operator_2=contains&filter_2=fertility&query=(Reproduction+OR+Fertility)+AND+(Ethiopia+OR+Tanzania+OR+Kenya+OR+Nigeria+OR+Sudan+OR+Somalia+OR+Eritrea+OR+Uganda))+AND+(Cow+OR+cattle)&scope = / https://cgspace.cgiar.org/discover?filtertype_1=title&filter_relational_operator_1=contains&filter_1=Cattle&filtertype_2=title&filter_relational_operator_2=contains&filter_2=reproduction&query=(Reproduction+OR+Fertility)+AND+(Ethiopia+OR+Tanzania+OR+Kenya+OR+Nigeria+OR+Sudan+OR+Somalia+OR+Eritrea+OR+Uganda))+AND+(Cow+OR+cattle)&scope = / https://cgspace.cgiar.org/discover?filtertype_1=title&filter_relational_operator_1=contains&filter_1=Cattle&filtertype_2=title&filter_relational_operator_2=contains&filter_2=reproductive&query=(Reproduction+OR+Fertility)+AND+(Ethiopia+OR+Tanzania+OR+Kenya+OR+Nigeria+OR+Sudan+OR+Somalia+OR+Eritrea+OR+Uganda))+AND+(Cow+OR+cattle)&scope = / https://cgspace.cgiar.org/discover?filtertype_1=title&filter_relational_operator_1=contains&filter_1=Cattle&filtertype_2=title&filter_relational_operator_2=contains&filter_2=fertility&query=(Reproduction+OR+Fertility)+AND+(Ethiopia+OR+Tanzania+OR+Kenya+OR+Nigeria+OR+Sudan+OR+Somalia+OR+Eritrea+OR+Uganda))+AND+(Cow+OR+cattle)&scope = /	10 (2)
Gates Open Research	(Reproduction OR Fertility OR reproductive) AND (Ethiopia OR Tanzania OR Kenya OR Nigeria) AND (Cow OR cattle Or bos)	0
PubMed	(((cow[Title/Abstract] OR cattle[Title/Abstract] or bos[Title/Abstract]) AND (reproduction[Title/Abstract] OR reproductive[Title/Abstract] OR fertility[Title/Abstract] OR "days open"[Title/Abstract] OR "calving age"[Title/Abstract] OR "calving interval"[Title/Abstract]) OR "days open"[Title/Abstract] OR "calving age"[Title/Abstract] OR "breeding age"[Title/Abstract] OR "non-return"[Title/Abstract] OR "conception rate"[Title/Abstract] OR "age at first calving"[Title/Abstract] OR "calving interval"[Title/Abstract])) AND (ethiopia OR kenya OR tanzania OR nigeria OR Sudan OR Somalia OR eritrea OR uganda) NOT (trial OR soil) AND ("2012/01/01"[Date—Create]: "3000"[Date—Create])	153 (36)
Research4life	https://hinari.summon.serialssolutions.com/#!/search?ho=t&include.ft.matches=f&rf=PublicationDate,2012-01-01:2022-07-06&l=en&q=(Ethiopia%20OR%20Tanzania%20OR%20Kenya%20OR%20Nigeria%20OR%20Sudan%20OR%20Somalia%20OR%20Uganda%20OR%20Eritrea)%20AND%20(Abstract:(%5C(Cow%20OR%20cattle%20Or%20bos%5C)%20AND%20%20%5C(Reproduction%20OR%20Fertility%20OR%20reproductive%5C)))	7 (1)
CAB Direct	(Reproduction OR Fertility OR reproductive) AND (Ethiopia OR Tanzania OR Kenya OR Nigeria OR Sudan OR Somalia OR Uganda OR Eritrea) AND (Cow OR cattle Or bos) yr:[2012 TO 2022]	183 (104)
Global ETD	(Reproduction OR Fertility OR reproductive) AND (Ethiopia OR Tanzania OR Kenya OR Nigeria OR Sudan OR Somalia OR Uganda OR Eritrea) AND (Cow OR cattle Or bos)	1 (1)

**Table 2:** – Results for each database and search string for bull fertility. For each search string, the first number of results is the number of returns identified; the second number is results after de-duplication

Database source	Search string	Number of results
Web of Science	(TS = (Reproduction) OR TS = (Fertility) OR TS = (reproductive) OR TS = (testicle) OR TS = (sperm)) AND ( TS = (Ethiopia) OR TS = (Tanzania) OR TS = (Kenya) OR TS = (Nigeria) OR TS = (Sudan) OR TS = (Somalia) OR TS = (Eritrea) OR TS = (Uganda)) AND TS = (bull) AND PY = (2011–2022) NOT (TS = (soil) OR TS = (trial) OR TS = (elephant) OR TS = (camel) OR TS = (giraffe) OR TS = (dog) OR TS = (rhinoceros))	14 (10)
Scopus	(TITLE-ABS-KEY(Reproduction) OR TITLE-ABS-KEY(Fertility) OR TITLE-ABS-KEY(reproductive) OR TITLE-ABS-KEY(testicle) OR TITLE-ABS-KEY(sperm) OR TITLE-ABS-KEY(semen))AND ( TITLE-ABS-KEY(Ethiopia) OR TITLE-ABS-KEY(Tanzania) OR TITLE-ABS-KEY(Kenya) OR TITLE-ABS-KEY(Nigeria) OR TITLE-ABS-KEY(Sudan) OR TITLE-ABS-KEY(Somalia) OR TITLE-ABS-KEY(Eritrea) OR TITLE-ABS-KEY(Uganda)) AND (TITLE-ABS-KEY(Bull)) AND PUBYEAR > 2011 AND NOT (TITLE-ABS-KEY(soil) OR TITLE-ABS-KEY(trial) OR TITLE-ABS-KEY(elephant) OR TITLE-ABS-KEY(camel) OR TITLE-ABS-KEY(giraffe) OR TITLE-ABS-KEY(dog) OR TITLE-ABS-KEY(rhinoceros))	27 (15)
Google Scholar	bull AND (Reproduction OR reproductive OR fertility OR Testicle OR sperm OR semen)[title] ((Ethiopia OR Tanzania OR Kenya OR Nigeria OR Sudan OR Somalia OR Uganda OR Eritrea))[key words] 2011–2022[PY]	72 (67)
CGIAR	https://cgspace.cgiar.org/discover?filtertype_1=title&filter_relational_operator_1=contains&filter_1=bull&query=(Reproduction+OR+Fertility+OR+testicle+OR+sperm+OR+semen)+AND+(Ethiopia+OR+Tanzania+OR+Kenya+OR+Nigeria+OR+Sudan+OR+Somalia+OR+Eritrea+OR+Uganda)+AND+(bull)&scope = /	17 (16)
Gates Open Research	(Reproduction OR Fertility OR reproductive OR sperm OR semen OR testicle) AND (Ethiopia OR Tanzania OR Kenya OR Nigeria OR Sudan OR Somalia OR Uganda OR Eritrea) AND (bull)	0
PubMed	(((bull[Title/Abstract]) AND ((reproduction[Title/Abstract] OR reproductive[Title/Abstract] OR fertility[Title/Abstract] OR testicle[Title/Abstract] OR semen[Title/Abstract]) OR sperm[Title/Abstract])) AND (ethiopia OR kenya OR tanzania OR nigeria OR Sudan OR Somalia OR eritrea OR uganda) NOT (trial[Title/Abstract] OR soil[Title/Abstract] OR elephant[Title/Abstract] OR camel[Title/Abstract] OR giraffe[Title/Abstract] OR dog[Title/Abstract] OR rhinoceros[Title/Abstract])) AND ("2012/01/01"[Date—Create]: "3000"[Date—Create])	10 (8)
Research4life	https://hinari.summon.serialssolutions.com/#!/search?ho=t&include.ft.matches=f&l=en&q=(bull)%20AND%20(ethiopia%20OR%20kenya%20OR%20tanzania%20OR%20nigeria%20OR%20Sudan%20OR%20Somalia%20OR%20eritrea%20OR%20uganda)%20AND%20(fertility%20OR%20sperm%20OR%20semen%20OR%20testicle%20OR%20reproduction%20OR%20reproductive)%20NOT%20(dog%20OR%20elephant%20OR%20camel%20OR%20soil%20OR%20giraffe%20OR%20rhinoceros)	192 (111)
CAB Direct	(Reproduction OR Fertility OR reproductive OR sperm OR semen OR testicle) AND (Ethiopia OR Tanzania OR Kenya OR Nigeria OR Sudan OR Somalia OR Uganda OR Eritrea) AND (bull) NOT (trial OR soil OR elephant OR camel OR giraffe OR dog OR rhinoceros) yr:[2012 TO 2023]	172 (72)
Global ETD	(Reproduction OR Fertility OR reproductive OR sperm OR semen OR testicle) AND (Ethiopia OR Tanzania OR Kenya OR Nigeria OR Sudan OR Somalia OR Uganda OR Eritrea) AND (bull) NOT (trial OR soil OR elephant OR camel OR giraffe OR dog OR rhinoceros)	3 (3)

### Study screening and selection

For all search strings in all databases, all studies retrieved were screened, first via title-abstract, and then full-text, following CEE guidelines (Collaboration for Environmental Evidence 2022) and by applying the pre-defined eligibility criteria. The inter-rater reliability of three reviewers (ISM, FKA and CS) was assessed, according to the selection criteria (Table 3), in a sample (10%) of study titles and abstracts using Fleiss’ Kappa statistic, in order to ensure cohesion and common understanding of the eligibility criteria. A Kappa score of 0.35 and 0.34 for female and male cattle, respectively, indicated fair agreement for inclusion*.* Where there were discrepancies or ambiguity in data, discussion between the reviewers was initiated until agreement was reached, including the decision to include or exclude unclear studies. Those studies considered relevant to the research question were then evaluated in full-text.

**Table 3:** Study selection criteria

Domain	Female cattle criteria	Male cattle criteria
Date range	2012–2022, inclusive	2012–2022, inclusive
Geographical scope	Ethiopia, Eritrea, Kenya, Nigeria, Somalia, South Sudan, Sudan, Tanzania and Uganda	Ethiopia, Eritrea, Kenya, Nigeria, Somalia, South Sudan, Sudan, Tanzania and Uganda
Type	Peer-reviewed journal articles, surveys, records, theses, conference proceedings Observational studies Original research	Peer-reviewed journal articles, surveys, records, theses, conference proceedings Observational and experimental (clinical trial) studies
Outcome	Measures of female cattle reproductive performance and fertility	Measures of male cattle (bull) reproductive performance and fertility
Language	English only	English only
Exclusions	Abstract or full-text unavailable Experimental (clinical trials) and modelling studies Literature reviews All other dates All other languages All other countries	Abstract or full-text unavailable Modelling studies Literature reviews All other dates All other languages All other countries Other bull species (camel, buffalo, bison, elephant, whale, walrus, crocodile, elk giraffe, hippopotamus, dogs)

Studies written in the English language between 2012 and 2022 were included, from nine countries; Ethiopia, Eritrea, Kenya, Nigeria, Somalia, South Sudan, Sudan, Tanzania and Uganda. Countries were purposely chosen to cover different agroecological zones and production systems and differently developed research provision in sub-Saharan East Africa and Nigeria. The search included peer-reviewed journal articles, data from published reports, theses, conference proceedings and field study reports. For female cattle, observational (cross-sectional and cohort) studies only were included. For bulls, observational studies were included, as well as experimental (clinical trials) studies. The latter were included due to the nature of observational studies in bulls, which typically only have very few bulls and therefore limited data. Modelling studies and literature reviews were excluded.

If abstracts or full-text studies were not available, they were excluded. Where possible, authors were contacted to request full-text studies. Where there was more than one form of an individual study’s results, the preferred version only was included to avoid duplication of data.

### Data management and extraction

Results of the database searches were downloaded as reference files and managed in CADIMA (Kohl et al. 2018), a free web tool facilitating the conduct of systematic reviews and maps. Full-text files were also assembled as a library in referencing software Mendeley (version 1.19.5, Elsevier, London, UK).

Bibliographic meta-data and study details were extracted from all studies considered for inclusion in the systematic map. Selected qualitative and quantitative metadata were extracted into a piloted Excel spreadsheet format. Bibliographic information extracted included study details such as author(s), citation, publication and publication year. Study details included country of study, locality, agroecological zone (AEZ), study period, season, production system, study type, sampling method, direction of sampling (prospective/retrospective), data source (e.g. records/questionnaire), cover (natural/artificial insemination), synchronisation, number of cattle, number of herds and sponsor. Population level details extracted were cattle breed, age and parity. A comments category for each type of data also allowed reviewers to record additional contextual information.

Outcome measures extracted for female cattle were: age at first service, age at first calving, calving to first service, calving to successful conception, calving interval, successful conception rate after insemination, number of services per conception, repeated breeding, culling due to infertility, number of pregnant adult cattle, and number of calving adult cattle. Due to the nature of measuring bull fertility, data extraction outcome measures were more generalised and included bull fertility, bull reproduction, testicle size, scrotal circumference, sperm (count, morphology, motility), semen, non-return rate, culling due to infertility, successful conception rate, number of services per conception, breeding soundness exam (BSE), and libido.

Where possible, data were collected in stratified ‘levels’ e.g. measures of fertility per breed, per parity or per production system. This level of detail depended on the individual studies*.* Following data extraction, the dataset was checked for consistency and data entry errors, referring back to relevant studies where required. Data were harmonised to allow for synthesis, by classifying data e.g. where specific breed data had been extracted, breed was classified into more generic groups such as ‘indigenous’ or ‘exotic’. For AEZ, we created groupings using the IFPRI (16) classification system (https://www.ifpri.org/publication/agro-ecological-zones-africa-south-sahara). In these instances, condensing the data into more manageable categories allowed for potential trends to be identified.

### Data synthesis

The evidence base identified within the systematic map is described in a narrative summary. The evidence base is also presented visually, as an interactive dashboard in the visualisation platform Tableau (Washington, USA) and can be accessed at https://www.livestockdata.org.

The quality of included studies was not appraised, as is typical in systematic mapping according to methodological guidance (James et al. 2016). Meta-data such as study design, sampling method, data source and publication were extracted, allowing for validity assessment of the included studies to be conducted in future systematic reviews.

## Results

### Female cattle

**Fig. 1:**
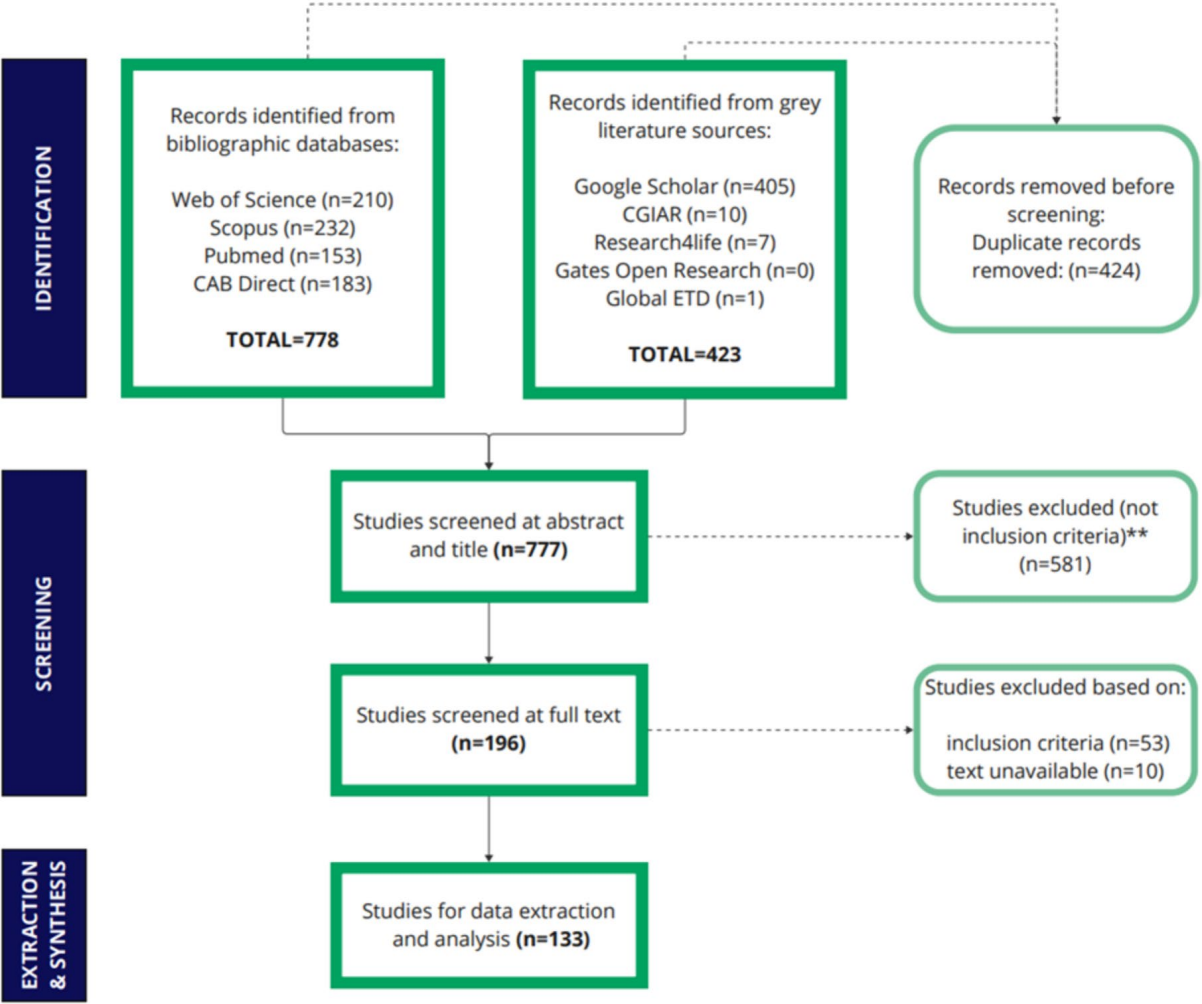
PRISMA flow diagram for female cattle study screening and inclusion (adapted from (Page et al. 2021))

The number of returns for each search are included in Table 1. Results of the literature searches are presented in Fig. 1. A total of 1,201 studies were identified (778 through databases and 423 from search engines and websites). Duplicates were removed (*n* = 424), and 581 studies were excluded based on their abstracts. Of the 196 full-text studies assessed for eligibility, 133 were included in the map (Supplementary File S2).

#### Distribution of studies

**Fig. 2:**
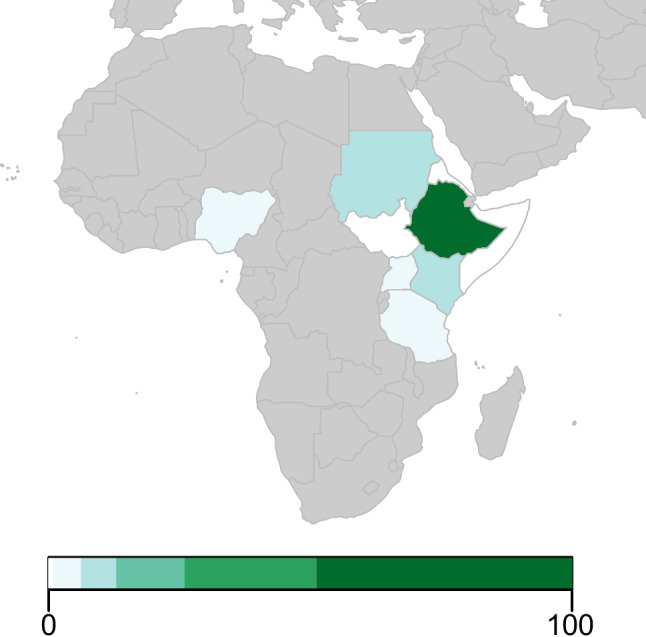
Number of retrieved female cattle studies per country

Included studies were predominantly conducted in Ethiopia (*n* = 100), followed by Kenya (*n* = 10), Sudan (*n* = 7), Nigeria (*n* = 6), Tanzania (*n* = 5), Uganda (*n* = 4) and Somalia (*n* = 1) (Fig. 2). No studies were found from Eritrea or South Sudan.

**Fig. 3:**
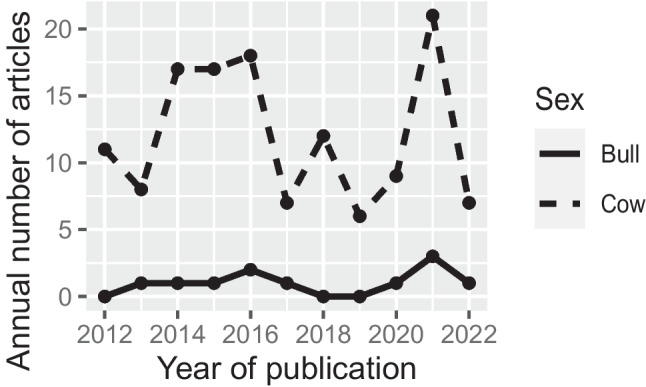
Number of retrieved studies published each year between 2012 and 2022

Figure 3 shows the number of studies published per year. While there was no remarkable trend, there were peaks in the numbers of studies published between 2014 and 2016, and in 2021 (*n* = 21). The number of published studies were considerably fewer in 2012–2013 and 2017–2020.

Of the 133 included studies, 54 did not specify the study period in which the work was carried out. Of the 79 studies that did specify study period, the studies ranged from 1960 to 2020. Journal articles were the most common type of publication (*n* = 122; 91.7%), followed by theses (*n* = 9; 6.8%) and conference proceedings (*n* = 2; 1.5%).

#### Parameters of female cattle reproductive performance

##### Age at first service

**Fig. 4:**
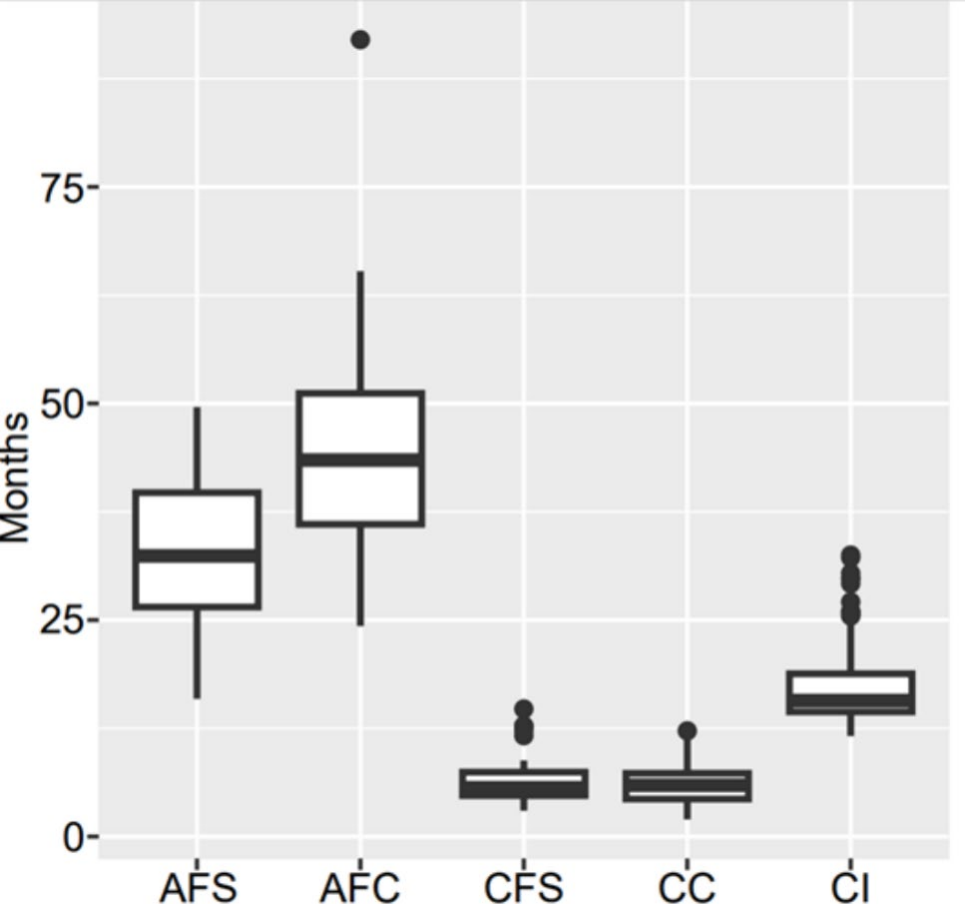
Range of values of reproductive performance parameters (that are reported in days or months). AFS = age at first service; AFC = age at first calving; CFS = calving to first service; CC = calving to successful conception; CI = calving interval

A total of 42 studies reported age at first service (AFS), from Ethiopia (*n* = 40), Kenya (*n* = 1) and Sudan (*n* = 1). The AFS reported ranged from 15.9–49.6 months (Fig. 4), where 15.9 was in Holstein Friesian crossbred and 49.6 was in local Fogera cattle. The lowest AFS was reported in an intensive commercial unit, and the highest was reported in a research centre. Overall, crossbred cattle tended to have lower AFS, local tended to have higher, and exotic cattle breeds were in between. In general, multiparous cattle tended to have lower AFS compared to primiparous.

##### Age at first calving

There were 38 studies that reported age at first calving (AFC), from Ethiopia (*n* = 36), Kenya (*n* = 1), and Sudan (*n* = 1). Reported AFC ranged from 24.3 months (Fig. 4) in Holstein Friesian crossbred cattle to 92 months in Holstein Zebu crossbred. The lowest AFC was reported in a large-scale dairy, and the highest was reported in a mixed crop-livestock system. Generally, crossbreeds had lower AFC than local or exotic breeds. Multiparous cattle generally had lower AFC compared to primiparous.

##### Calving to first service

A total of 11 studies reported calving to first service (CFS), all from Ethiopia. Reported CFS ranged from 89 days (Fig. 4) in local dairy cattle to 442 days in Horro and Horro-Jersey crossbreeds. The shortest CFS was reported in a mixed crop-livestock system and the longest was in a research centre and smallholder farms. Overall, crossbred cattle tended to have shorter CFS, local breeds had longer, and exotic breeds were in between. There was no trend in parity and CFS observed.

##### Calving to successful conception

Calving to conception interval (CC) was reported in 35 studies, from Ethiopia (*n* = 31), Somalia (*n* = 1), Sudan (*n* = 1), Tanzania (*n* = 1) and Uganda (*n* = 1). Reported CC ranged from 59 days (Fig. 4) in local dairy cattle to 366 days in Boran crossbreeds. The shortest CC was observed in a dairy system and the longest in an intensive smallholder dairy system. Despite the shortest and longest intervals described above, overall, crossbred cattle tended to have shorter CC, local tended to have longer and exotic breeds were in between. Multiparous cattle tended to have shorter CC compared to primiparous.

##### Calving interval

Calving interval (CI) was reported in 86 studies, from Ethiopia (*n* = 71), Kenya (*n* = 7), Nigeria (*n* = 2), Somalia (*n* = 1), Sudan (*n* = 2) and Tanzania (*n* = 3). The shortest CI was 11.6 months (Fig. 4) reported in Holstein Friesian and crossbreed cattle, and the longest CI of 32.5 months reported in Holstein Friesian crossbreeds. The shortest CI was reported in a commercial dairy, and the longest was observed in mixed crop-livestock. In general, crossbred cattle tended to have shorter CI and local breeds tended to have longer. Overall, CI tended to gradually reduce with increasing parity, from parity two onwards.

##### Conception rate

Conception rate (CR) was reported in five studies, from Ethiopia (*n* = 4) and Nigeria (*n* = 1), ranging from 41.8–69%, with both lowest and highest in crossbred or local Zebu cattle. In general, multiparous cattle had lower CR compared to primiparous. There was no trend in production system and CR.

##### Number of services per conception

A total of 74 studies reported number of services (NS), from Ethiopia (*n* = 62), Kenya (*n* = 4), Nigeria (*n* = 4), Sudan (*n* = 2), Somalia (*n* = 1) and Tanzania (*n* = 1). Reported NS ranged from 1 in smallholder dairy cattle in Kenya to 3.78 in dairy cattle in Lume district, Ethiopia. There was no consistent association of breed with NS, with studies reporting both higher (e.g., Belay and Chakravarty 2014; Kumar et al. 2014) and lower (e.g., (Duguma 2021)) NS in local breeds compared to crossbred cows. Similarly, studies examining the effect of parity on NS reported varying results.

##### Repeat breeding

A total of 28 studies reported repeat breeding, from Ethiopia (*n* = 24), Kenya (*n* = 3) and Sudan (*n* = 1), ranging from 0.6% in intensive/ semi-intensive and extensive dairy, to 61.7% in smallholder dairy (mixed crop-livestock subsistence). The study reporting 61.7% stated that repeat breeding was commonly reported in herds using AI solely. Very few studies reported on associations of breed, production systems or parity with number or proportion of repeated breeders; however, several studies reported associations of disease (e.g., bovine viral diarrhoea (Asmare et al. 2018; Aragaw et al. 2021) and infectious bovine rhinotracheitis (Tadeg et al. 2021)).

##### Culling due to infertility

Eight studies reported culling due to infertility, from Ethiopia (*n* = 4), Uganda (*n* = 2), Kenya (*n* = 1) and Nigeria (*n* = 1). Age at culling and percentage culling were typically reported, across production systems.

#### Parameters per study

**Fig. 5:**
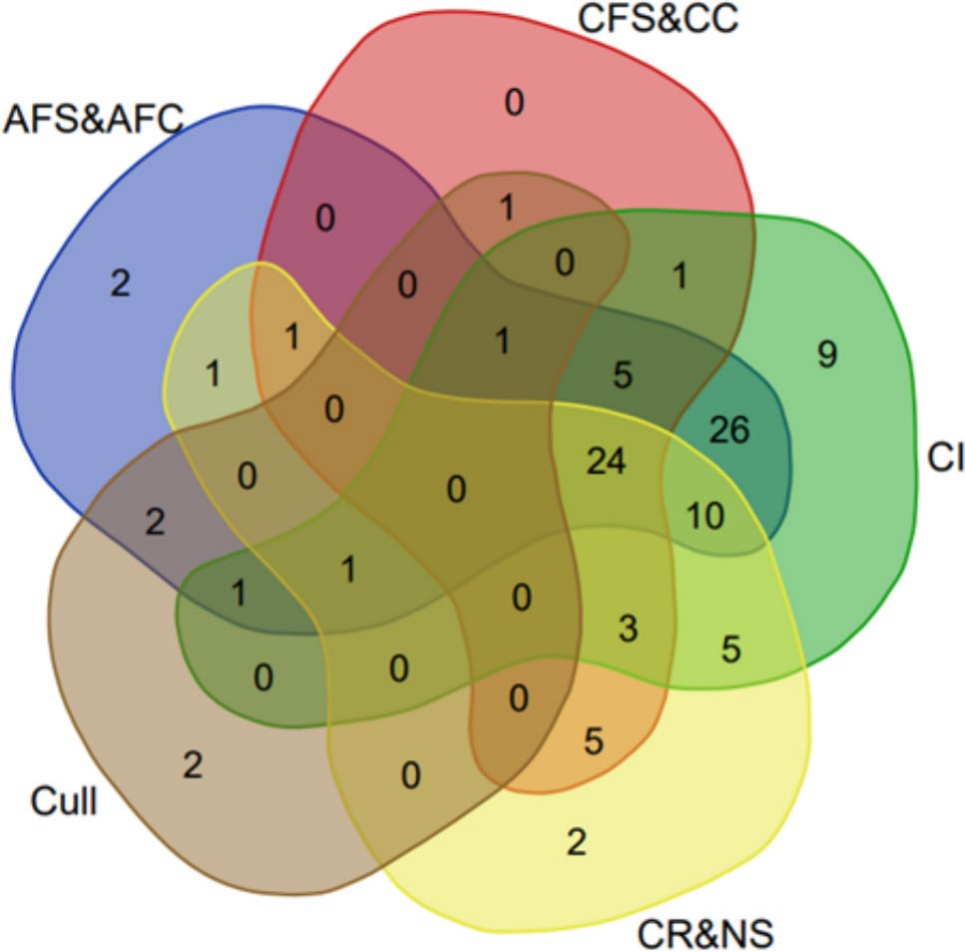
Venn diagram showing parameters per study. Each parameter is depicted by a colour, and where they overlap indicates how many studies reported that combination. AFS = age at first service; AFC = age at first calving; CFS = calving to first service; CC = calving to successful conception; CI = calving interval; CR = conception rate; NS = number of services; Cull = culling due to infertility

The number of parameters reported in each study varied, as shown in Fig. 5; some studies reported a single parameter, for example nine studies reported only calving interval, and two studies reported only age at first service and or age at first calving. No studies reported all parameters, but 24 studies reported four parameters.

### Bulls

**Fig. 6:**
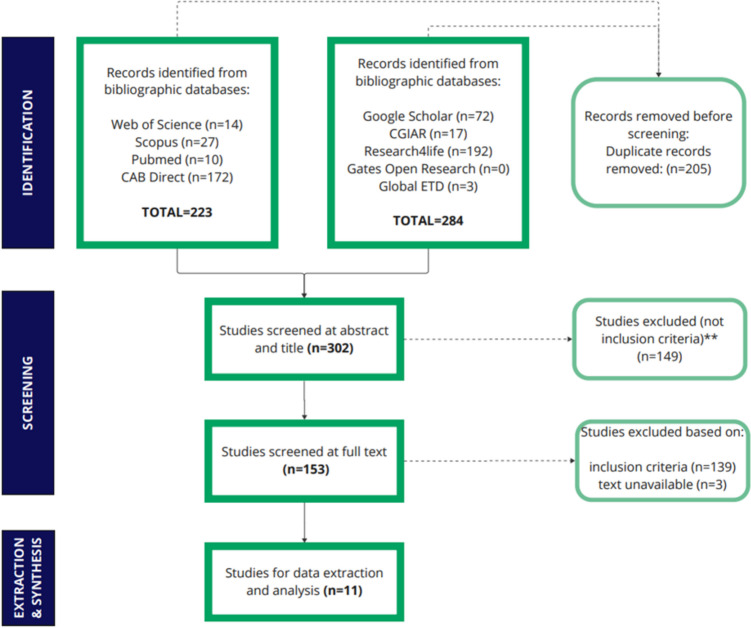
PRISMA flow diagram for bull study screening and inclusion (adapted from (Page et al. 2021))

The number of returns for each search is included in Table 2. Results of the literature searches are presented in Fig. 6. A total of 507 studies were identified (223 through databases and 284 from search engines and websites). Duplicates were removed (*n* = 205), and 149 studies were excluded based on their abstracts. Of the 153 full-text studies assessed for eligibility, 11 were included in the map (Supplementary File S3).

#### Distribution of studies

**Fig. 7:**
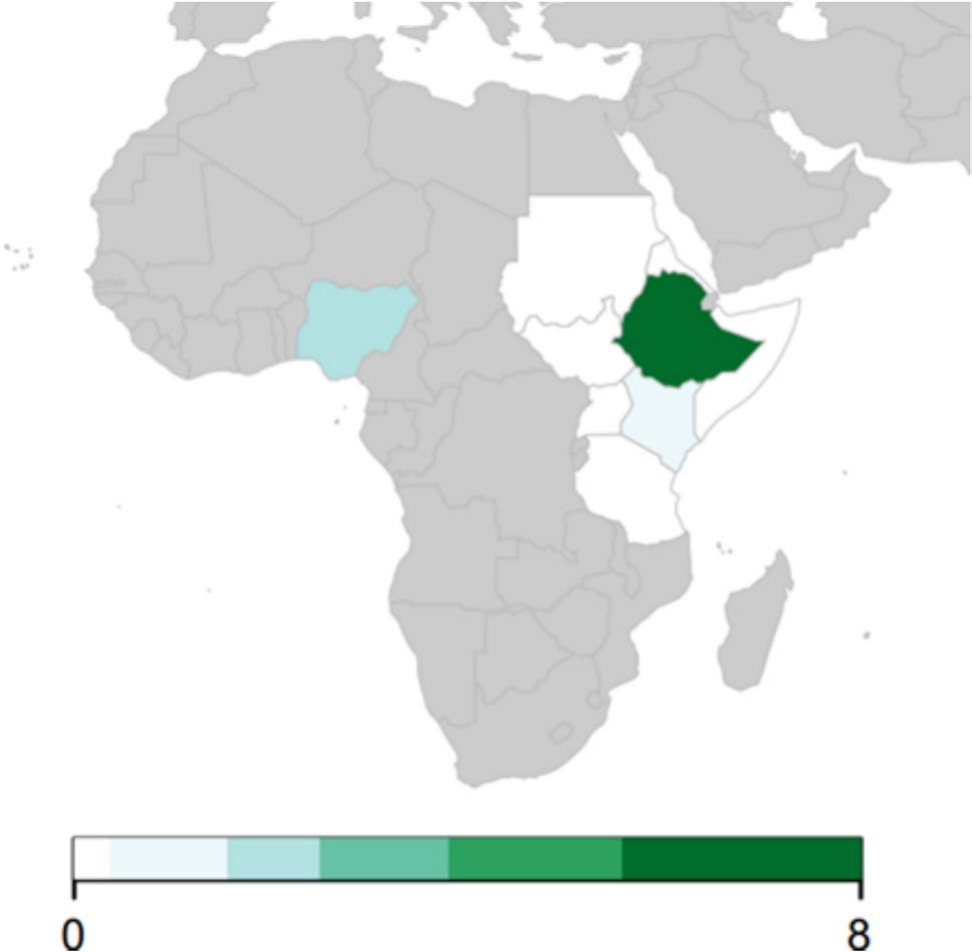
Number of retrieved bull studies per country

Most studies were conducted in Ethiopia (*n* = 8), followed by Nigeria (*n* = 2), and Kenya (*n* = 1) (Fig. 7).

Studies were published between 2013 and 2022, with the exceptions of 2018 and 2019 (Fig. 3). Most years had a single publication, except 2021 which had three studies published. Of the 11, four studies did not specify the study period in which the work was carried out. Of the seven that did specify year, the study periods ranged from 2005 to 2016. All of the studies were journal articles. Four of the 11 studies did not report production system.

#### Parameters of bull reproductive performance

Three studies reported testicular abnormalities, and seven studies reported semen/sperm characteristics. One reported on breeding soundness and one on testicular circumference. Most within-study comparisons were between different breeds.

## Discussion

Overall, primary research in female cattle reproductive performance appears to be relatively well reported, predominantly in journal articles, with some reported in theses and conference proceedings. The majority of studies were from Ethiopia, which was not surprising given the high number of agricultural learning institutes, a large population of livestock, and the focus of funding and research that has taken place here. Few or no studies were from Eritrea, Somalia and South Sudan, which may reflect the lack of research resources in these countries. Fewer studies than expected were identified from Kenya and Tanzania; with the International Livestock Research Institute (ILRI) operating in these countries, we expected there to be a greater number of studies. It may be that historically this has been an area of research there, but that the dates of our systematic map (2012–2022) did not capture older studies. Additionally, relatively few studies identified were from Nigeria and Uganda. The searches used in this systematic map were limited to English language and it is possible that studies written in other local languages were not captured. For those countries that do not appear to have an abundance of data, we question whether fertility is recognised as a problem, or can we infer that surrounding countries have similar reproductive performance? This is a large assumption and highly speculative, as conditions and management practices vary greatly even within countries which could result in varied reproductive performance.

Studies reported a range of female cattle reproductive performance parameters, the most common being CI and NS. Many studies reported only one or few parameters, although a reasonable number of studies reported multiple parameters. However, despite reporting multiple parameters, studies generally did not provide an overview of parameters combined, with no explanation for parameter values, which makes interpretation of the parameters challenging. Few studies reported culling due to infertility, which is an important measure of reproductive performance and herd health. Several studies reported that multiparous cattle tended to have lower AFS compared to primiparous; while this sounds nonsensical, we believe the studies meant *year* rather than *parity* per se.

A comparatively small number of studies were identified for bull reproductive performance. Given that a “bull is half the herd”, the reproductive performance of the individual bull is a crucial factor in determining the reproductive performance of cows, yet the fertility of bulls is rarely investigated, at least when they are used in natural service (Ball and Peters 2004a). This systematic map identified only 11 published studies on bull fertility, indicating a huge gap in evidence in this area. This may in part be due to data availability, for example there is likely dairy genetics data that artificial insemination companies hold, but due to their private nature it is not published and, therefore, not accessible. Within the limited literature, the concept of bull reproductive performance was lacking, with a lack of discussion on factors such as how to measure and compare reproductive performance, and how do feed and work affect fertility. There are only few parameters to measure reproductive performance in bulls compared to female cattle, with a ‘normal’ bull described as having “at least 30% sperm motility, 70% normal sperm morphology, and a minimum scrotal circumference based on age” (Hopkins and Spitzer 1997). In addition, bull behaviour and management can affect fertility (BVA, n.d.).

Sound reproductive performance is an essential component of cattle production and management (Ball and Peters 2004b). It has been said that “if you wish to know the state of affairs on your farm, key figures are essential…you compare them with the key figures for previous periods…Key figures also tell you whether you can do better than you’re doing now” (Hulsen 2008). Whilst the purpose of a systematic map is to present the type and quantity of evidence, rather than describing the research findings (Saran and White 2018), we wanted to briefly comment on the data, specifically the parameter values. Overall, the female cattle parameter values appear to be reasonable, indicating that it is possible to have good reproductive performance in LMICs. There is a lot of variation in the data and it is difficult to know the level of genuine variability versus uncertainty; the study results are all possible values. Whilst there are ‘typical’ parameter values described in the literature, for example AFC 22–26 months, CFS 50–70 days, CC 85–115 days, CI 365 days (Ball and Peters 2004b; Hudson et al. 2012), there will be variation depending on the climatic conditions of the study area, farming systems and breed. Whilst some of the values suggest poor reproductive performance, it is important to consider the natural variation and that ‘typical’ values vary depending on the setting and context i.e. what might be a ‘target’ value in one setting may be completely unachievable or unsuitable for another. These parameter values all represent study results so can be considered valid values, useful as modelling boundaries.

As described in the methodology, after the data extraction process, data were harmonised to allow for synthesis, which involved classifying data, for example where breed data had been extracted, breed was classified into more generic groups such as ‘indigenous’ or ‘exotic’, and we used the IFPRI (16) classification system to group agroecological zones (AEZ) for all studies, where reported. There was a great amount of such metadata missing from studies, frequently making it impossible to classify. For example, studies often reported multiple AEZ but without specific associated stratified data, often making it impossible to identify associations in reproductive performance parameters and metadata such as AEZ.

### Limitations

Relating to missing data and challenges in classification and misclassification, is the lack of established agreed definitions (ontologies) for metadata, such as AEZ, production system and breed types. This makes synthesis and comparisons of results challenging. These limitations support the need for reporting standards, such as the STROBE statement to improve reporting in veterinary observational studies (Sargeant and O’Connor 2014). Another limitation was the inadvertent omission of some studies. While many studies were identified in the search results, there is the possibility that more could have been identified if further databases were included, or if snowballing was applied. As the study was mapping evidence from nine LMICs, financial barriers may prevent publishing in peer-reviewed journals, and non-English language studies would not have been captured, resulting in potential selection bias. While authors were contacted to request missing full texts, only two of 12 cow studies and one of four bull study requests were responded to, resulting in potential self-selection bias.

## Conclusion

This systematic map provides the recent published evidence on reproductive performance in cattle in Eritrea, Ethiopia, Kenya, Nigeria, Somalia, South Sudan, Sudan, Tanzania and Uganda. We identified a relative abundance of research in cattle reproductive performance from Ethiopia, and a general gap in studies on bull reproductive performance. This review is intended to inform further primary research and policy- and decision-makers, by identifying evidence gaps. The interactive dashboard provides an accessible place for stakeholders to visualise the available evidence, and it is hoped that the gaps in this important but somewhat neglected area of research receive more stakeholder attention in efforts to improve cattle production.

## Supplementary Information

Below is the link to the electronic supplementary material.

Supplementary file1 (PDF 353 KB)

Supplementary file2 (PDF 556 KB)

Supplementary file3 (PDF 426 KB)

## Data Availability

The datasets generated during the study are available in online repository. https://dataverse.harvard.edu/dataset.xhtml?persistentId=doi:10.7910/DVN/VIHOXM
