# Supplementary material for: Systematic map of recent evidence on reproductive performance of cattle in Africa
Source: Trop Anim Health Prod. 2024 Jul 22;56(7):218. doi: 10.1007/s11250-024-04074-z (PMC11271395; doi:10.1007/s11250-024-04074-z)
Supplement: Supplementary file 2 — Supplementary file2 (PDF 556 KB) [file 11250_2024_4074_MOESM2_ESM.pdf]

Supplementary File S2

Table S1. Cow studies included in systematic map.

Reference	Full citation
(Abaya et al., 2021)	Abaya, G., Kebede, A. and Jilo, K., 2021. A Comparative Study on Prevalence of Major Reproductive Health Problems of Indigenous and Cross Breed Dairy Cattle in Jimma Zone, South Western Ethiopia. Animal and Veterinary Sciences, 9, 39
(Abebe and Demissie, 2021)	Abebe, K. and Demissie, T., 2021. Assessment on Milk Yield and Reproductive Performances of Crossbred Dairy Cattle in Selected Dairy Production Systems of Central Highlands of Ethiopia. Asian Journal of Dairy and Food Research
(Abraha and Yousuf, 2020)	Abraha, B. and Yousuf, J., 2020. Effect of artificial insemination on the productive and reproductive performance of dairy cattle in smallholder farmers, Laelay maichew district, Tigray, Ethiopia. International Journal of Agriculture and Biosciences, 9, 83–87
(Adane et al., 2021)	Adane, Z., Yemane, N. and Hidosa, D., 2021. Reproductive and Productive Performance of Indigenous Cattle Breed in Bena-Tsemay District of SoutOmo, South-Western Ethiopia. Journal of Fisheries & Livestock Production, 9, 1000312
(Alemayehu, 2021)	Alemayehu, M.M., 2021. Evaluation of Genetic and Non-Genetic Factors on Growth and Reproductive Performance of Fogera Cattle at Chagni Ranch, North West Ethiopia. (Haramaya University)
(Ali et al., 2013)	Ali, T., Lemma, A. and Yilma, T., 2013. Reproductive performance of dairy cows under artificial insemination in south and northwest part of Ethiopia. Livestock Research for Rural Development, 25
(Ali et al., 2015)	Ali, T., Lemma, A. and Yilma, T., 2015. Effect of Management Practices on Reproductive Performance of Smallholder Dairy Cattle. Austin Journal of Veterinary Science & Animal Husbandry, 2, 1–5
(Aragaw et al., 2018)	Aragaw, K., Sibhat, B., Ayelet, G., Skjerve, E., Gebremedhin, E.Z. and Asmare, K., 2018. Seroprevalence and factors associated with bovine viral diarrhea virus (BVDV) infection in dairy cattle in three milksheds in Ethiopia. Tropical Animal Health and Production, 50, 1821–1827
(Aragaw et al., 2021)	Aragaw, K., Regassa, F., Sibhat, B., Abayneh, T., Gelaye, E., Deresse, G., Egan, S. and Asmare, K., 2021. Seroprevalence and association of bovine viral diarrhea virus (BVDV) serostatus with reproductive problems in dairy cattle in central and southern Ethiopia. Tropical Animal Health and Production, 53, 1–9
(Asgedom et al., 2016)	Asgedom, H., Damena, D. and Duguma, R., 2016. Seroprevalence of bovine brucellosis and associated risk factors in and around Alage district, Ethiopia. SpringerPlus, 5, 1–8
(Asmare et al., 2013)	Asmare, K., Regassa, F., Robertson, L.J. and Skjerve, E., 2013. Seroprevalence of Neospora caninum and associated risk factors in intensive or semi-intensively managed dairy and breeding cattle of Ethiopia. Veterinary Parasitology, 193, 85–94
(Asmare et al., 2018)	Asmare, K., Sibhat, B., Ayelet, G., Gebremedhin, E.Z., Lidete, K.A. and Skjerve, E., 2018. Serological evidence of Bovine herpesvirus-1, Bovine Viral Diarrhea virus and Schmallenberg virus infections in relation to reproductive disorders in dairy cattle in Ethiopia. Acta Tropica, 178, 236–241
(Assaminew and Ashenafi, 2015)	Assaminew, S. and Ashenafi, M., 2015. Feed formulation and feeding impact on the performance of dairy cows in Central Highland of Ethiopia. Livestock Research for Rural Development, 27
(Assemu, 2015)	Assemu, T.S., 2015. Estimation of Genetic and Non-genetic parameter for growth and reproductive performance traits and designing conservation strategies for Fogera cattle. (Bahir Dar University, Ethiopia)
(Ayalew et al., 2013)	Ayalew, T., Duguma, B. and Tolemariam, T., 2013. Prevalence of cattle diseases and productive and reproductive traitsof cattle in Ilu Aba Bora zone, south western Ethiopia. Global Veterinaria, 10, 614–619
(Bashir and El Zubeir, 2013)	Bashir, H. and El Zubeir, I., 2013. Milk Production and Reproduction Performance of Baggara Cattle Raised Under Extensive and Semi-Extensive Systems in South Kordofan State, Sudan. Journal of Animal Production Advances, 3, 192
(Belay and Chakravarty, 2014)	Belay, B. and Chakravarty, A.K., 2014. Genetic analyses of early-expressed reproduction traits of Boran and their crosses with Holstein Friesian and Jersey in Central Highlands of Ethiopia. Tropical Animal Health and Production, 46, 113–119
(Belay, 2016)	Belay, D.L., 2016. Assessment of Reproductive Performance of Local and Crossbred Dairy Cattle in Sidama Zone, Southern Ethiopia. Journal of Natural Sciences Research, 6, 16–22

(Beneberu et al., 2021)	Beneberu, N., Alemayehu, K., Mebratie, W., Getahun, K. and Tesema, Z., 2021. Evaluation of Reproductive Performance of Jersey Cattle Raised under Semi-intensive Management in Ethiopia. Asian Journal of Dairy and Food Research, 40, 8–13
(Benti and Zewdie, 2014)	Benti, A.D. and Zewdie, W., 2014. Major reproductive health problems of indigenous Borena cows in Ethiopia. Journal of Advanced Veterinary and Animal Research, 1, 182–188
(Bereda et al., 2014)	Bereda, A., Yilma, Z. and Nurfeta, A., 2014. Dairy production system and constraints in Ezha districts of the Gurage zone, Southern Ethiopia. Global Veterinaria, 12, 181–186
(Birhanu et al., 2015)	Birhanu, T., Mohammed, T., Kebede, K. and Tadesse, M., 2015. Productive and Reproductive Performances of Ethiopian Boran Cattle with Different Levels of Holstein Friesian Inheritance. American-Eurasian Journal of Scientific Research, 10, 278–286
(Bisrat and Nigussie, 2016)	Bisrat, M. and Nigussie, H., 2016. Comparative evaluation on productive and reproductive performance of indigenous and crossbred dairy cow managed under smallholder farmers in Endamehoni District, Tigray, Ethiopia. Journal of Biology, Agriculture and Healthcare, 6, 96–100
(Chalchissa et al., 2014)	Chalchissa, G., Mekasha, Y. and Urge, M., 2014. Reproductive performance of crossbred dairy cattle in selected urban and peri-urban farms of mid rift valley, Ethiopia. African Journal of Agricultural Research, 9, 1687–1693
(Chaters et al., 2018)	Chaters, G., Rushton, J., Dulu, T.D. and Lyons, N.A., 2018. Impact of foot-and-mouth disease on fertility performance in a large dairy herd in Kenya. Preventive Veterinary Medicine, 159, 57–64
(Chebo et al., 2012)	Chebo, C., Wuletawb, Z. and Ayalewc, W., 2012. Reproduction Performance and Milk Off-Take of Indigenous Cattle of Gamo Goffa Zone, Southern-Western Ethiopia. Ethiopian Journal of Animal Production, 12, 73–86
(DeLay et al., 2020)	DeLay, N.D., Thumbi, S.M., Vanderford, J., Otiang, E., Ochieng, L., Njenga, M.K., Palmer, G.H. and Marsh, T.L., 2020. Linking calving intervals to milk production and household nutrition in Kenya. Food Security, 12, 309–325
(Deresa et al., 2020)	Deresa, B., Tulu, D. and Deressa, F.B., 2020. Epidemiological Investigation of Cattle Abortion and Its Association with Brucellosis in Jimma Zone, Ethiopia. Veterinary Medicine: Research and Reports, 11, 87–98
(Destaw and Kefyalew, 2018)	Destaw, W.M. and Kefyalew, A.W., 2018. Evaluation of the reproductive performance of Holstein Friesian dairy cows in Alage ATVET college, Ethiopia. International Journal of Livestock Production, 9, 131–139
(Dinka, 2012)	Dinka, H., 2012. Reproductive performance of crossbred dairy cows under smallholder condition in Ethiopia. International Journal of Livestock Production, 3, 563
(Dinka, 2013)	Dinka, H., 2013. The major reproductive disorders of dairy cows in and around Asella town, Central Ethiopia. Journal of Veterinary Medicine and Animal Health, 5, 113–117
(S. Diro et al., 2021)	Diro, S., Mamo, T., Getahun, W., Yohannis, A., Mebratu, T. and Musemma, R., 2021. Farmers' Perception of Dairy Cattle Reproductive Performance in the Central Highlands of Ethiopia. Ethiopian Journal of Animal Production, 21, 1–18
(Samuel Diro et al., 2021)	Diro, Samuel, Mamo, T., Getahun, W., Mebratu, T. and Musemma, R., 2021. Economic Loss of Dairy Cattle Poor Reproductive Performance in Central Highlands of Ethiopia. Advances in Dairy Research, 9, 1–12
(Ducrotoy et al., 2016)	Ducrotoy, M.J., Majekodunmi, A.O., Shaw, A.P.M., Bagulo, H., Musa, U.B., Bertu, W.J., Gusi, A.M., Ocholi, R.A., Bryssinckx, W. and Welburn, S.C., 2016. Fulani cattle productivity and management in the Kachia Grazing Reserve, Nigeria. Pastoralism, 6, 1–19
(Duguma, 2020)	Duguma, B., 2020. A survey of management practices and major diseases of dairy cattle in smallholdings in selected towns of Jimma zone, south-western Ethiopia. Animal Production Science, 60, 1838–1849
(Duguma, Kechero, et al., 2012)	Duguma, B., Kechero, Y. and Janssens, G.P.J., 2012. Productive and reproductive performance of Zebu X Holstein-Friesian crossbred dairy cows in Jimma town, Oromia, Ethiopia. Global Veterinaria, 8, 67–72
(Duguma, Tegegne, et al., 2012)	Duguma, B., Tegegne, A. and Hegde, B.P., 2012. Smallholder livestock production system in Dandi district, Oromia Regional State, central Ethiopia. Global Veterinaria, 8, 472–479
(Duguma, 2021)	Duguma, B., 2021. Productive and reproductive performance of crossbred and indigenous dairy cows at smallholdings in selected towns of Jimma Zone, Ethiopia. Animal Production Science, 61, 92–100

(Elamin et al., 2012)	Elamin, K.M., Elebead, R.A., Mohammed, S.A. and Musa, A.M., 2012. Some Productive and Reproductive Traits of Kenana × Friesian Cattle in Sudan. World's Veterinary Journal, 2, 49–53
(Elemam and Nekheila, 2012)	Elemam, M.B. and Nekheila, A.M.A., 2012. Some productive traits of crossbred dairy cows in the farm of University of Khartoum, Sudan. Emirates Journal of Food and Agriculture, 24, 155–159
(Elfaki, 2015)	Elfaki, M.A.M., 2015. Productive, Reproductive Performance and Body Measurements of Butana Cattle in Atbara Research Station, Animal Production Research Co-orporation, River Nile State, Sudan. (Univeristy of Gezira)
(Elhassan et al., 2016)	Elhassan, A.M., Babiker, A.M., Ahmed, M.E. and El Hussein, A.M., 2016. Coinfections of Sudanese dairy cattle with bovine herpes virus 1, bovine viral diarrhea virus, bluetongue virus and bovine herpes virus 4 and their relation to reproductive disorders. Journal of Advanced Veterinary and Animal Research, 3, 332–337
(Elhassan et al., 2015)	Elhassan, A.M., Fadol, M.A., Elfahal, A.M.A. and El Hussein, A.R.M., 2015. A cross sectional study on reproductive health disorders in dairy cattle in Sudan. Journal of Advanced Veterinary and Animal Research, 2, 101–106
(Gabriel, 2022)	Gabriel, M.S., 2022. Brucellosis infection dynamics in cattle and the impacts on production and reproduction in pastoral settings of Tanzania. Journal of Veterinary Medicine and Animal Health, 14, 38–43
(Gai et al., 2022)	Gai, A.B., Wahome, R.G. and Bett, R.C., 2022. Impact of resettlement on livestock production and performance among the Maasai pastoralists of RAPland village, Olkaria Kenya. Pastoralism, 12, 1–7
(Garoma, 2014)	Garoma, S., 2014. Reproductive and productive performance of Kereyu Sanga cattle in Fentalle District of Oromia Region, Ethiopia. Journal of Cell and Animal Biology, 8, 28–33
(Gebrekidan et al., 2016)	Gebrekidan, B., Tegegne, A. and Regassa, F., 2016. Assessment of reproductive performance of Begait cattle in in-situ and ex-situ sites and in different production systems in northern Ethiopia. Animal Reproduction Science, 166, 1–8
(Gebremeskel et al., 2019)	Gebremeskel, A.K., Tanga, B.M., Nigatu, Y. and Olkeba, C.F., 2019. Reproductive health problems and associated risk factors in intensively managed dairy cows in Alage, Southern Ethiopia. Journal of Veterinary Medicine and Animal Health, 11, 12–16
(Genzebu et al., 2016)	Genzebu, D., Tamir, B. and Berhane, G., 2016. Study of Reproductive and Production Performance of Cross Breed Dairy Cattle under Smallholders Management System in Bishoftu and Akaki Towns. International Journal of Advanced Research in Biological Sciences, 3, 118–123
(Getahun, 2018)	Getahun, K., 2018. Genetic and Non-Genetic Parameter Estimation for Productive and Reproductive Performances of Crossbred Dairy Cattle At Holetta Research Center. (Haramaya University)
(Getenet et al., 2014)	Getenet, A., Berhanu, M. and Desie, S., 2014. Major postpartum problems of dairy cows managed in small and medium scale production systems in Wolaita Sodo, Ethiopia. African Journal of Agricultural Research, 9, 2775–2780
(Gizachew, 2019)	Gizachew, N., 2019. Major Reproductive Health and Productive Problems of Dairy Cattle in Selected Dairy Farms in Bishoftu, Central Ethiopia. International Journal of Sciences, 5, 6–10
(Gurmessa et al., 2015)	Gurmessa, K., Tolemariam, T., Tolera, A., Beyene, F. and Demeke, S., 2015. Productive and Reproductive Performance of Horro Cattle and Dairy Product Utilization by Smallholder Farmers. American-Eurasian Journal of Scientific Research, 10, 361–367
(Guta, 2021)	Guta, D., 2021. Evaluation of reproduction performance and calf sex ratio of dairy cattle in selected locations of South-East Oromia. International Journal of Agricultural Science and Food Technology, 7, 170–192
(Hadush et al., 2013)	Hadush, A., Abdella, A. and Regassa, F., 2013. Major prepartum and postpartum reproductive problems of dairy cattle in Central Ethiopia. Journal of Veterinary Medicine and Animal Health, 5, 118–123
(Hailay, 2013)	Hailay, A., 2013. Evaluation of the Reproductive and Artifical Insemination Service Efficiency of Dairy Cattle in Eastern Zone of Tigray, North Ethiopia. (Haramaya University)
(Haile et al., 2014)	Haile, A., Tsegaye, Y. and Tesfaye, N., 2014. Assessment of Major Reproductive Disorders of Dairy Cattle in Urban and Per Urban Area of Hosanna, Southern Ethiopia. Animal and Veterinary Sciences, 2, 135
(Hamid et al., 2021)	Hamid, M., Abduraman, S. and Tadesse, B., 2021. Risk Factors for the Efficiency of Artificial Insemination in Dairy Cows and Economic Impact of Failure of First Service Insemination in and around Haramaya Town, Oromia Region, Eastern Ethiopia. Veterinary Medicine International, 1–6

(Hansar et al., 2014)	Hansar, E., Lemma, A. and Yilma, T., 2014. Pre-service ultrasonic and manual evaluation of the reproductive organs of dairy cows presumed to be in estrus. SpringerPlus, 3, 1–5
(Hassan et al., 2020)	Hassan, H.M., Dubad, A.B., Muse, M.M., Ali, A.M. and Ali, B.S., 2020. Assessment of Reproductive Efficiency and Herd Dynamics of Local Cattle Breeds in Benadir Region, Somalia. Advances in Animal and Veterinary Sciences, 8, 1100–1108
(Hunde et al., 2015)	Hunde, D., Mészáros, G., Dessie, T., Assefa, G., Tadesse, M. and Sölkner, J., 2015. Milk yield and reproductive performance of pure jersey dairy cattle in the central highlands of Ethiopia. Livestock Research for Rural Development, 27
(Hundie et al., 2013)	Hundie, D., Beyene, F. and Duguma, G., 2013. Early Growth and Reproductive Performances of Horro Cattle and thier F1 Jersey Crosses in and around Horro-Guduru Livestock Production and Research Center, Ethiopia. Science, Technology and Arts Research Journal, 2, 134
(Hussein, 2018)	Hussein, T., 2018. Productive and Reproductive Performance of Indigenous Ethiopian Cow under Small Household Management in Dawro Zone, Southern Ethiopia. International Journal of Current Research and Academic Review, 6, 35–41
(Kakooza et al., 2018)	Kakooza, S., Tumwebaze, M., Nabatta, E., Byaruhanga, J., Tayebwa, D.S. and Wampande, E., 2018. Risk Factors and Co-Existence of Infectious Causes of Reproductive Failures in Selected Uganda Cattle and Goats: A Brucella spps-Toxoplasma gondii Study. Open Access Library Journal, 05, 1–12
(Kanuya et al., 2015)	Kanuya, N., Shoo, J., Mtera, Y., Mshana, J., Barongo, A. and Basile, M., 2015. Reproductive performance of artificially inseminated dairy cows under smallholder production system in selected areas of Rwanda and Tanzania. Tanzania Veterinary Journal, 29, 62–70
(Kanyima et al., 2014)	Kanyima, B., Bage, R., Owiny, D., Ntallaris, T., Lindahl, J., Magnusson, U. and Nassuna-Musoke, M., 2014. Husbandry Factors and the Resumption of Luteal Activity in Open and Zero-Grazed Dairy Cows in Urban and Peri-Urban Kampala, Uganda. Reproduction in Domestic Animals, 49, 673–678
(Kassahun, 2016)	Kassahun, M.M., 2016. Milk production and reproductive performance of local and crossbreed dairy cows in selected districts of West Gojam Zone, Amhara Region, Ethipia. (Bahir Dar University)
(Kebede et al., 2016)	Kebede, D., Alemayehu, K. and Girma, E., 2016. Reproductive and Productive Performance of Fogera Cattle in Lake Tana Watershed, North Western Amhara, Ethiopia. Journal of Reproduction and Infertility, 6, 56–62
(Kebede, 2015)	Kebede, H., 2015. Productive and reproductive performance of holstein-friesian cows under farmer's management in Hossana town, Ethiopia. International Journal of Dairy Science, 10, 126–133
(Kebede et al., 2017)	Kebede, H., Jimma, A., Getiso, A. and Zelke, B., 2017. Characterization of Gofa Cattle Population, Production System, Production and Reproduction Performance in Southern Ethiopia. Journal of Fisheries & Livestock Production, 05, 1000237
(Kidane et al., 2019)	Kidane, A.B., Delesa, K.E., Mummed, Y.Y. and Tadesse, M., 2019. Reproductive and Productive Performance of Holstein Friesian and Crossbreed Dairy Cattle at Large, Medium and Small Scale Dairy Farms in Ethiopia. International Journal of Advanced Research in Biological Sciences, 6, 15–29
(Kifle and Moges, 2016)	Kifle, M. and Moges, N., 2016. Major Reproductive Health Disorders of Cow in and Around Gondar, North West Ethiopia. Journal of Reproduction and Infertility, 7, 88–93
(Kubkomawa et al., 2017)	Kubkomawa, I.H., Adamu, M.S., Ogundu, M., Okoli, I. and Udedibie, A., 2017. Socio-cultural, Herd Structure and Reproductive Practices of Pastoral Cattle Producers in Adamawa State, Nigeria. International Journal of Animal Research, 1
(Kugonza et al., 2012)	Kugonza, D.R., Nabasirye, M., Hanotte, O., Mpairwe, D. and Okeyo, A.M., 2012. Pastoralists' indigenous selection criteria and other breeding practices of the long-horned Ankole cattle in Uganda. Tropical Animal Health and Production, 44, 557–565
(Kumar et al., 2014)	Kumar, N., Tkui, K. and Bisrat, A., 2014. Reproductive performance of dairy cows under farmer's management in and around Mekelle, Ethiopia. Livestock Research for Rural Development, 26
(Lemma and Negussu, 2015)	Lemma, A. and Negussu, S., 2015. Effect of musculoskeletal disorders on the reproductive performance of Holstein Zebu cross dairy cows. Journal of Biology, Agriculture and Healthcare, 5, 22–25
(Mai et al., 2014)	Mai, H.M., Voh Jr., A.A. and Deshi, P.S., 2014. Some fertility indices in an artificially inseminated Bunaji and Bokoloji herds in Kaduna state, Nigeria. Global Veterinaria, 12, 171–175
(Mai et al., 2015)	Mai, H.M., Irons, P.C. and Thompson, P.N., 2015. Brucellosis, genital campylobacteriosis and other factors affecting calving rate of cattle in three states of Northern Nigeria. BMC Veterinary Research, 11

(Majekodunmi et al., 2016)	Majekodunmi, A.O., Dongkum, C., Langs, T., Shaw, A. and Welburn, S., 2016. Improved productivity and sustainable pastoral systems in an era of insecurity—Fulani herds of the southern Jos Plateau, North-Central Nigeria. Tropical Animal Health and Production, 48, 1719–1728
(Mathew, 2017)	Mathew, C.M., 2017. Infections associated with reproductive disorders in cattle in Tanzania: occurrence, characterization and impact. (Norwegian University of Life Sciences)
(Meaza, 2017)	Meaza, M., 2017. Longitudinal Study on Reproductive and Productive Performances of Smallholder Crossbred Dairy Cattle in and Around Wolaita Sodo, Snnpr, Ethiopia. Journal of Veterinary Science & Technology, 08, 1000458
(Mekonnen et al., 2012)	Mekonnen, A., Haile, A., Dessie, T. and Mekasha, Y., 2012. On farm characterization of Horro cattle breed production systems in western Oromia, Ethiopia. Livestock Research for Rural Development, 24
(Mekonnin, 2017)	Mekonnin, A.B., 2017. Monitoring and improving reproductive performance of crossbred dairy cattle in Tigray Region, Ethiopia. (University of Edinburgh)
(Mekonnin et al., 2015)	Mekonnin, A.B., Harlow, C.R., Gidey, G., Tadesse, D., Desta, G., Gugssa, T. and Riley, S.C., 2015. Assessment of Reproductive Performance and Problems in Crossbred (Holstein Friesian X Zebu) Dairy Cattle in and Around Mekelle, Tigray, Ethiopia. Animal and Veterinary Sciences, 3, 94
(Mengistu et al., 2016)	Mengistu, D.W., Wondimagegn, K.A. and Demisash, M.H., 2016. Reproductive performance evaluation of holstein friesian and their crosses with boran cattle breeds in ardaita agricultural technical vocational education training college dairy farm, Oromia Region, Ethiopia. Iranian Journal of Applied Animal Science, 6, 805–814
(Mezgebe et al., 2018)	Mezgebe, G., Gizaw, S. and Urge, M., 2018. Growth, reproductive, and productive performance of Begait cattle under different herd management systems in northern Ethiopia. Tropical Animal Health and Production, 50, 1313–1318
(Misebo et al., 2018)	Misebo, F., Gashaw, T. and Yilma, M., 2018. Assessment on major reproductive health problems of dairy cattle in Boloso Sore, Southern Ethiopia. Journal of Veterinary Medicine and Animal Health, 10, 224–230
(Mitiku et al., 2022)	Mitiku, M., Megersa, B. and Sheferaw, D., 2022. Major reproductive disorders and seroprevalence of brucellosis in dairy cows of Kembata-Tembaro zone, Southern Ethiopia. Ethiopian Veterinary Journal, 26, 1–17
(Muasya et al., 2014)	Muasya, T.K., Peters, K.J. and Kahi, A.K., 2014. Effect of diverse sire origins and environmental sensitivity in Holstein-Friesian cattle for milk yield and fertility traits between selection and production environments in Kenya. Livestock Science, 162, 23–30
(Mujibi et al., 2014)	Mujibi, F.D.N., Ojango, J., Rao, J.E.O., Karanka, T., Kihara, A., Marete, A., Baltenweck, I., Poole, J., Rege, J.E.O., Gondro, C., Weerasinghe, W.M.S.P., Gibson, J.P. and Okeyo, A.M., 2014. Use of High Density SNP Genotypes to Determine the Breed Composition of Cross Bred Dairy Cattle in Smallholder Farms: Assessment of Reproductive and Health Performance. In:, Proceedings of 10th World Congress on Genetics Applied to Livestock Production.,
(Mungube et al., 2014)	Mungube, E.O., Njarui, D.M.G., Gatheru, M., Kabirizi, J. and Ndikumana, J., 2014. Reproductive and health constraints of dairy cattle in the peri-urban areas of semi-arid eastern Kenya. Livestock Research for Rural Development, 26
(Mungube et al., 2019)	Mungube, E.O., Njarui, D.M.G., Maichomo, M.W., Olum, M.O., Ndirangu, P.N., Kabirizi, J., Ndikumana, J. and Mwangi, G., 2019. Reproductive performance indicators of dairy cattle in selected small-scale dairy farms in semi-arid Eastern Kenya. Livestock Research for Rural Development, 31
(Mwambene et al., 2014)	Mwambene, P.L., Chawala, A., Illatsia, E., Das, S.M., Tungu, B. and Loina, R., 2014. Selecting indigenous cattle populations for improving dairy production in the Southern Highlands and Eastern Tanzania. Livestock Research for Rural Development, 26
(Njoroge et al., 2021)	Njoroge, E.N., Mutembei, H.M., Kipyegon, A.N., Kimeli, P. and Olum, M.O., 2021. Prevalence of Repeat Breeding Syndrome in Dairy Cattle in Selected Regions of Kenya. International Journal of Veterinary Science, 10, 114–118
(Olum et al., 2020)	Olum, M., Mungube, E., Nakami, W., Kidali, J., Njenga, E., Maichomo, M., Tsuma, V. and Mugambi, J., 2020. A Cross-sectional Study on Infertility and its Causes in Small Holder Dairy Cattle in Selected Counties of Kenya. International Journal of Veterinary Science, 9, 534–539
(Opoola et al., 2020)	Opoola, O., Banos, G., Ojango, J.M.K., Mrode, R., Simm, G., Banga, C.B., Beffa, L.M. and Chagunda, M.G.G., 2020. Joint genetic analysis for dairy cattle performance across countries in sub-Saharan Africa. South African Journal of Animal Science, 50, 507–520
(Regassa and Kacho, 2019)	Regassa, M. and Kacho, B.B., 2019. Study on Major Reproductive Health Problems of Dairy Cattle at Mekele. Journal of Advanced Research in Agriculture Science and Technology, 2, 36–42
(Sarba et al., 2016)	Sarba, E.J., Getaneh, A.M. and Borena, B.M., 2016. Seroprevalence and Associated Risk Factors of Brucellosis in Dairy Cattle in Selected Towns of West Shewa, Ethiopia. Bulletin of Animal Health and Production in Africa, 64, 387–395

(Senbeta et al., 2021)	Senbeta, E.K., Ameha Zeleke, N. and Taffa Dingidie, G., 2021. Impact of feed optimization and extension intervention on productive and reproductive performance in commercial dairy farms of urban areas in the eastern region of Ethiopia Indian Journal of Dairy Science, 74, 266–270
(Sendeku et al., 2016)	Sendeku, A., Kumar, D., Abegaz, S. and Mekuriaw, G., 2016. Evaluations of Reproductive Performances of Fogera Cattle Breed in Selected Districts of Amhara Region, Ethiopia. International Journal of Pharma Medicine and Biological Sciences, 5, 52
(Seyoum et al., 2014)	Seyoum, K., Mengistu, A., Melesse, K. and Melesse, A., 2014. Assessment of Reproductive and Productive Performance of Dairy Cattle Breeds in Three Districts of East Shoa Zone, Central Ethiopia. In:, Proceedings of the 21st Annual Conference of the Ethiopian Society of Animal Production, held in Addis Ababa, Ethiopia, August 28-30 2013.,
(Sibhat et al., 2018)	Sibhat, B., Ayelet, G., Gebremedhin, E.Z., Skjerve, E. and Asmare, K., 2018. Seroprevalence of Schmallenberg virus in dairy cattle in Ethiopia. Acta Tropica, 178, 61–67
(Siyoum et al., 2016)	Siyoum, T., Yohannes, A., Shiferaw, Y., Asefa, Z. and Eshete, M., 2016. Major reproductive disorders on Jersey breed dairy cattle at Adea Berga dairy farm, West Shewa Zone, Oromia Region, Ethiopia. Ethiopian Veterinary Journal, 20, 91–103
(Stephen et al., 2022)	Stephen, J., Paul, B.T., Ibrahim, M.M., Bukar, M.M. and Mshelia, G.D., 2022. Serological Evidence of Leptospira hardjo Antibodies and The Incidence of Reproductive Disorders in Selected Smallholder Cattle and Goat Farms from Maiduguri, Nigeria. Journal of Advanced Veterinary Research, 12, 1–5
(Sulieman et al., 2017)	Sulieman, M.S., Makawi, S.E.A. and Ibrahim, & K.E.E., 2017. Association between postpartum blood levels of glucose and urea and fertility of cross-bred dairy cows in Sudan. South African Journal of Animal Science, 47, 595–605
(Swai et al., 2014)	Swai, E.S., Mollel, P. and Malima, A., 2014. Some factors associated with poor reproductive performance in smallholder dairy cows: The case of Hai and Meru districts, northern Tanzania. Livestock Research for Rural Development, 26
(Tadeg, Biru, et al., 2021)	Tadeg, W.M., Biru, A.L., Mekonnen, T.Y. and Reda, A.A., 2021. Dairy cattle fertility and the role of estrus induction in improving reproductive performance of cows with no history of estrus after calving. Livestock Research for Rural Development, 33
(Tadeg, Lemma, et al., 2021)	Tadeg, W.M., Lemma, A., Yilma, T., Asgedom, H. and Reda, A.A., 2021. Seroprevalence of infectious bovine rhinotracheitis and brucellosis and their effect on reproductive performance of dairy cattle. Journal of Veterinary Medicine and Animal Health, 13, 106–113
(B. Tadesse et al., 2022)	Tadesse, B., Reda, A.A., Kassaw, N.T. and Tadeg, W., 2022. Success rate of artificial insemination, reproductive performance and economic impact of failure of first service insemination: a retrospective study. BMC Veterinary Research, 18, 226
(M. Tadesse et al., 2022)	Tadesse, M., Getahun, K., Hunde, D. and Gelmessa, U., 2022. Analysis of the Non-genetic Factors Influencing the Performance of High-grade and Inter se Mated Crossbred Dairy Cows at Holetta Dairy Research Farm, Ethiopia. Asian Journal of Dairy and Food Research, 41, 38–42
(Taye et al., 2012)	Taye, M., Belihu, K., Bekana, M. and Sheferaw, D., 2012. Assessment of impacts of tsetse and trypanosomosis control measures on cattle herd composition and performance in southern region, Ethiopia. Tropical Animal Health and Production, 44, 1759–1763
(Tayebwa et al., 2015)	Tayebwa, D., Bigirwa, G., Byaruhanga, J. and Kasozi, K., 2015. Prevalence of Endometritis and Its Associated Risk Factors in Dairy Cattle of Central Uganda. American Journal of Experimental Agriculture, 7, 155–162
(Tedla et al., 2018)	Tedla, M., Mehari, F. and Kebede, H., 2018. A cross-sectional survey and follow up study on major dairy health problems in large and small scale urban farms in Mekelle, Tigray, Ethiopia. BMC Research Notes, 11
(Tekle et al., 2016)	Tekle, Z., Guadu, T., Demissie, K., Mitku, F. and Demessie, Y., 2016. Assessment of Reproductive Performance of Crossbred Dairy Cattle among Dairy Farms in and Around Addis Ababa, Central Ethiopia. Global Veterinaria, 17, 358–364
(Temesgen et al., 2022)	Temesgen, Y.M., Assen, A.A., Gizaw, T.T., Minalu, B.A. and Mersha, A.Y., 2022. Factors affecting calving to conception interval (days open) in dairy cows located at Dessie and Kombolcha towns, Ethiopia. PloS one, 17, e0264029
(Terefe et al., 2021)	Terefe, E., Belay, G. and Admasu, E., 2021. Reproductive performance of crossbred dairy cattle under semiintensive management system in Arsi highland, Ethiopia. Arsi Journal of Science and Innovation, 6, 1–19
(Tesfaye et al., 2015)	Tesfaye, A., Alemayehu, L., Tefera, Y. and Endris, A., 2015. Factors affecting the reproductive performance of smallholder dairy cows in two regions of Ethiopia. Livestock Research for Rural Development, 27
(Tigicho et al., 2012)	Tigicho, T., Belihu, K. and Terefe, G., 2012. Impacts of tsetse challenge on herd composition and mortality, lactation and reproductive performance of cattle in Dawuro Zone of Southern Region, Ethiopia. Revue de Médecine Vétérinaire, 163, 405–410

(Tolosa et al., 2021)	Tolosa, F., Netsere, M. and Habtamu, Y., 2021. Assessment of Major Reproductive Disorders in Dairy Cattle in and around Bale Robe, Oromia Regional State, Ethiopia. Veterinary Medicine International, 8855718, 1–8
(Tschopp, Conlan, et al., 2021)	Tschopp, R., Conlan, A.J.K., Gemechu, G., Almaw, G., Hattendorf, J., Zinsstag, J. and Wood, J.L.N., 2021. Effect of Bovine Tuberculosis on Selected Productivity Parameters and Trading in Dairy Cattle Kept Under Intensive Husbandry in Central Ethiopia. Frontiers in Veterinary Science, 8, 1–10
(Tschopp, Gemechu, et al., 2021)	Tschopp, R., Gemechu, G. and Wood, J.L.N., 2021. A Longitudinal Study of Cattle Productivity in Intensive Dairy Farms in Central Ethiopia. Frontiers in Veterinary Science, 8, 1–11
(Tsega et al., 2020)	Tsega, M., Tadesse, Y. and Urge, M., 2020. Production and Reproduction Performance of Simada Cattle Population in Three Districts of North-Western Ethiopia. Global Journal of Animal Scientific Research, 8, 37–43
(Walelign, 2018)	Walelign, N., 2018. Productive and Reproductive Performance of Crossbred Dairy Cattle at Menkorer Agro Industry Enterprise Dairy Farm Debre Markos Ethiopia. (Haramaya University)
(Wario et al., 2017)	Wario, H.T., Roba, H.G., Aufderheide, M. and Kaufmann, B., 2017. Reproductive performance and herd growth potentials of cattle in the Borana pastoral system, southern Ethiopia. Animal Production Science, 57, 161–169
(Wassie et al., 2015)	Wassie, T., Mekuriaw, G. and Mekuriaw, Z., 2015. Reproductive Performance for Holstein Friesian x Arsi and Holstein Friesian x Boran Crossbred Cattle. Iranian Journal of Applied Animal Science, 5, 35–40
(Weldegebriel, 2015)	Weldegebriel, D.G., 2015. Assessment of Production and Reproductive Performances of Cattle and Husbandry Practices in Bench-Maji Zone, Southwest Ethiopia. Global Journal of Animal Scientific Research, 3, 441–452
(Weldeslasse et al., 2012)	Weldeslasse, G.T., Zeleke, Z.M. and Gangwar, S., 2012. Reproductive and Productive Performance of Dairy Cattle in Central Zone of Tigray, Northern Ethiopia. International Journal of Advanced Biological Research, 2, 58–63
(Woldeyohannes, 2020)	Woldeyohannes, T., 2020. Assessment of Husbandry practices, Production and Reproductive Performance of Indigenous Cattle in Hadiya Zone, Southern Ethiopia. International Research Journal of Science and Technology, 1, 177–198
(Worku et al., 2016)	Worku, D., Alemayehu, K. and H/Melekote, M., 2016. Comparative reproductive performance evaluation of Holstein Friesian cattle breeds in two different agro ecological conditions, Oromia region, Ethiopia. Animal Genetic Resources, 58, 31–42
(Yimam et al., 2021)	Yimam, K., Chimde, L. and Fekata, A., 2021. Assessment of Dairy Cattle Productive and Reproductive Performance in West Guji Zone, Oromia Regional State, Ethiopia. Journal of Indigenous Knowledge and Development Studies, 3, 1–16
(Yitagesu et al., 2021)	Yitagesu, E., Jackson, W., Kebede, N., Smith, W. and Fentie, T., 2021. Prevalence of bovine abortion, calf mortality, and bovine viral diarrhea virus (BVDV) persistently infected calves among pastoral, peri-urban, and mixed-crop livestock farms in central and Northwest Ethiopia. BMC Veterinary Research, 17
(Yohannes and Alemu, 2019)	Yohannes, B. and Alemu, B., 2019. Reproductive Disorders in Dairy Cattle; Retrospective Study in Asella Town, Central Ethiopia. Journal of Dairy & Veterinary Sciences, 9, 1–6
(Zereu and Lijalem, 2016)	Zereu, G. and Lijalem, T., 2016. Production and Reproduction Performances of Local Dairy Cattle: In the Case of Rural Community of Wolaita Zone, Southern Ethiopia. Journal of Fisheries & Livestock Production, 4, 1000176
