# Supplementary material for: Systematic map of recent evidence on reproductive performance of cattle in Africa
Source: Trop Anim Health Prod. 2024 Jul 22;56(7):218. doi: 10.1007/s11250-024-04074-z (PMC11271395; doi:10.1007/s11250-024-04074-z)
Supplement: Supplementary file 3 — Supplementary file3 (PDF 426 KB) [file 11250_2024_4074_MOESM3_ESM.pdf]

Supplementary File S3

Table S2. Bull studies included in systematic map.

Reference	Full citation
(Alemu et al., 2015)	Alemu, J., Hassen, M., Pal, M. and Agegnehu, B., 2015. Comparative studies on semen for gross and microscopic abnormalities in bulls at preservice evaluation time. Haryana Veterinarian, 53, 135–138
(Amare et al., 2016)	Amare, E., Habtemariam, K., Samuel, A., Shimelis, D. and Belina, D., 2016. Incidence of gross pathological conditions of the reproductive organs in bulls. Indian Journal of Veterinary Pathology, 40, 305–311
(Ayodeji and Suwaiba, 2013)	Ayodeji, A.A. and Suwaiba, W., 2013. Cryptorchidism among indigenous breeds of bulls in a semi-arid region of Nigeria. Macedonian Veterinary Review Vet Rev, 36, 123–128
(Eshetu Gemeda, 2017)	Eshetu Gemeda, A., 2017. Gross testicular abnormalities in indigenous breeds of bulls in Eastern Ethiopia. Journal of Advanced Veterinary and Animal Research, 4, 200–206
(Goshme et al., 2021)	Goshme, S., Asfaw, T., Demiss, C. and Besufekad, S., 2021. Evaluation of motility and morphology of frozen bull semen under different thawing methods used for artificial insemination in North Shewa zone, Ethiopia. Heliyon, 7, e08183 (Elsevier)
(Iliyasu et al., 2015)	Iliyasu, D., Munir, A.S., Auwal, U., O.S., O., Mustapha, R.A. and Ahmed, I., 2015. Consequences of Chronic Dermatophilosis on Semen Quality and Reproductive Performance of Friesian Bull in Multipurpose Farm in Zari. International Journal of Livestock Research, 5, 46–53
(Mutembei et al., 2016)	Mutembei, H.M., Origa, R. and Agumbah, G.J.O., 2016. An In-Vitro Criterion for Comparative Assessment of Fertility Characteristics of Bull Semen in Kenya. Journal of Agricultural Science and Food Technology, 2, 100–104
(Reda et al., 2020)	Reda, A.A., Almaw, G., Abreha, S., Tadeg, W. and Tadesse, B., 2020. Bacteriospermia and Sperm Quality of Cryopreserved Bull Semen Used in Artificial Insemination of Cows in South Wollo Zone, Ethiopia. Veterinary Medicine International, 2020, 1–11
(Seyoum and Lemma, 2022)	Seyoum, K. and Lemma, A., 2022. Breeding soundness of semen producing bulls, their semen quality and inter relationship of functional tests with spermatozoa motility percentage. Journal of Veterinary Medicine and Animal Health, 14, 1–11
(Seyoum et al., 2021a)	Seyoum, K., Lemma, A. and Tera, A., 2021a. Effect of breed, age and period of production on bovine semen quality used for artificial insemination. International Journal of Livestock Production, 12, 43–48
(Seyoum et al., 2021b)	Seyoum, K., Lemma, A. and Tera, A., 2021b. Influence of breed on motility and motion characteristics of fresh, chilled and frozen bull spermatozoa. International Journal of Livestock Production, 12, 37–42
